# Stratifying atherosclerotic cardiovascular disease by SMuRF burden in a Middle-Eastern country: A multiregistry study of demographics, comorbidities, and therapeutic trends

**DOI:** 10.1371/journal.pone.0332302

**Published:** 2025-09-22

**Authors:** Osama Alkouri, Zainab Albikawi, Ahmad Rajeh Saifan, Haitham Khatatbeh, Anas Ababneh, Alanoud Alobaidly, Omar Qaladi, Abdulhafith Alharbi, Abdullah Hasan, Mohamad Jarrah, Mohammad Abuadas, Ayman Hammoudeh, Nader Alotaibi, Mohannad E. AbuRuz, Fadwa Alhalaiqa, Nezam Al-Nsair

**Affiliations:** 1 Faculty of Nursing, Yarmouk University, Irbid, Jordan; 2 Nursing Department, Yarmouk University, Irbid, Jordan; 3 College of Nursing and Health Sciences, Flinders University, Adelaide, Australia; 4 Community and Psychiatric Mental Health, King Saud University, Riyadh, Saudi Arabia; 5 College of Nursing, University of Hail, Hail, Saudi Arabia; 6 College of Nursing, The Public Authority for Applied Education and Training, Safat, Kuwait; 7 Department of Internal Medicine, Faculty of Medicine, Jordan University of Sciences and Technology, Irbid, Jordan; 8 Department of Cardiology, Istishari Hospital, Amman, Jordan; 9 College of Nursing, King Saud University, Riyadh, Saudi Arabia; 10 Psychiatric/Mental Health Department, Pre-Clinical Affairs/College of Nursing, Qatar University, Doha, Qatar; 11 Hind Bint Maktoum College of Nursing and Midwifery, Mohammed Bin Rashid University of Medicine and Health Science, Dubai Health, Dubai, United Arab Emirates; The University of Jordan, JORDAN

## Abstract

**Background:**

Atherosclerotic cardiovascular disease (ASCVD) is a growing public health challenge in the Middle East, driven by increasing rates of obesity, diabetes, and hypertension. However, limited region-specific data exist on the distribution and impact of Standard Modifiable Risk Factors (SMuRFs) among affected individuals. Most available evidence is derived from Western populations, which may not reflect the unique demographic, cultural, and epidemiological profiles of Middle Eastern communities.

**Objective:**

This study aims to compare demographic profiles, clinical presentations, comorbidities, and pharmacologic management among Middle Eastern patients with ASCVD, stratified by SMuRF burden: SMuRF-less (no risk factors), 1–2 SMuRFs, and 3–4 SMuRFs. The goal is to identify clinically relevant differences across these groups and address the significant gap in region-specific data on ASCVD risk factors and outcomes.

**Methods:**

Data were pooled from six established cardiovascular registries and the Jordan SMuRF-less patient study. Baseline characteristics, cardiovascular risk profiles, comorbidities, use of secondary prevention therapies, and one-year outcomes were analyzed across the three SMuRF categories.

**Results:**

Among 5,540 patients, the group with 3–4 SMuRFs exhibited the highest prevalence of hypertension (88.9%), diabetes (35.4%), smoking (54.0%), and a family history of cardiovascular disease (39.3%). This group also showed increased rates of chronic kidney disease (7.3%) and heart failure (15.1%). Statin, aspirin, and beta-blocker use increased in parallel with SMuRF burden. Key lipid profiles deteriorated with increasing SMuRFs, with the highest LDL cholesterol observed in patients with 1–2 SMuRFs.

**Conclusions:**

This study provides valuable insight into the clinical characteristics and management patterns of Middle Eastern patients with atherosclerotic cardiovascular disease (ASCVD), stratified according to the burden of standard modifiable risk factors (SMuRFs). An increased SMuRF burden was associated with a higher prevalence of comorbid conditions, including hypertension, diabetes mellitus, dyslipidemia, chronic kidney disease, and heart failure. Utilizing data from six regional registries, this study represents the first comprehensive, region-specific analysis of the role of modifiable risk factors in this population. The findings underscore the critical need for individualized, risk-based prevention and management strategies to mitigate the rising burden of ASCVD across the Middle East.

## Introduction

Atherosclerotic cardiovascular disease (ASCVD) remains a leading global health burden, caused by the accumulation of lipids, inflammatory cells, and fibrotic tissue within the arterial intima. This process leads to the formation of atherosclerotic plaques, which, when ruptured or eroded, can precipitate major cardiovascular events such as myocardial infarction and ischemic stroke [1]. According to the World Health Organization (WHO), cardiovascular diseases (CVDs) account for over 30% of global deaths, with ASCVD contributing significantly to this figure [2]. The prevalence of ASCVD is particularly alarming, as it is the leading cause of mortality worldwide, responsible for an estimated 18 million deaths annually [3–5]. In the Middle East, the burden of ASCVD is particularly pronounced due to a combination of lifestyle factors, genetic predispositions, and limited access to healthcare. Individuals in this region tend to develop ASCVD at younger ages compared to global averages, largely due to the high prevalence of diabetes, hypertension, and dyslipidemia [6–8]. One study found that approximately one in five adults with type 2 diabetes in the Middle East had cardiovascular disease, with many cases attributed to ASCVD [9]. In Jordan specifically, recent data show a rising prevalence of ASCVD, with cardiovascular diseases ranking among the leading causes of death—largely driven by smoking, physical inactivity, and unhealthy diets [10,11].

Key modifiable cardiovascular risk factors collectively referred to as standard modifiable risk factors (SMuRFs)—include hypertension, type 2 diabetes mellitus, elevated low-density lipoprotein (LDL) cholesterol, and tobacco use [12]. These factors not only play a central role in the pathogenesis of ASCVD but also form the foundation for its prevention and management [13]. The presence of even one SMuRF significantly increases the risk of coronary artery disease (CAD) and its complications, such as acute coronary syndrome (ACS), stroke, and peripheral arterial disease (PAD). The Framingham Heart Study was among the first to establish the pivotal role of SMuRFs, which have since been validated as robust predictors of ASCVD risk in numerous subsequent studies [8,14]. Individuals with diabetes are considered to have ASCVD risk equivalents, meaning their risk is comparable to those with established cardiovascular disease [15]. Moreover, the interplay between risk factors such as the frequent co-occurrence of hypertension and diabetes—can amplify their individual effects, further increasing cardiovascular risk [15].

Stratifying individuals based on SMuRF burden provides an effective framework for risk assessment. Those without modifiable risk factors termed SMuRF-less are generally at lower risk for ASCVD [16]. However, a notable proportion of ASCVD events occur in this group, revealing limitations in conventional risk factor-based assessment models [17–19]. Some studies suggest that clinical outcomes for SMuRF-less patients may be as favorable as, or even better than, those with one or more risk factors [20–22]. Conversely, individuals with one or two SMuRFs face a higher risk of ASCVD. Even a single factor, such as hypertension or hypercholesterolemia, significantly increases the likelihood of cardiovascular events. These patients often present with higher GRACE risk scores, reflecting more severe clinical profiles [22]. The incidence of acute myocardial infarction (AMI) also increases with SMuRF burden, particularly among individuals with abnormal lipoprotein(a) levels [23].

Patients with multiple SMuRFs are at the highest risk and require comprehensive management strategies [23–25]. SMuRFs form the basis of risk assessment tools such as the Pooled Cohort Equations, which estimate 10-year ASCVD risk and guide preventive interventions. However, these models may not fully capture ASCVD risk in diverse populations, where social determinants of health—such as socioeconomic status, education, and access to care—play a critical role [26,27]. The prevalence of SMuRF-less individuals varies widely by population. In a study of 8,680 Asian patients, only 8.6% were SMuRF-less, compared to 10.5% in the United States, 14.5% in Canada, 14.9% in Sweden, and 19–25% in Australia [28]. Notably, 50% of AMI patients had at least one modifiable risk factor, highlighting their central role in ASCVD pathogenesis [29]. The proportion of individuals with three or more SMuRFs is even higher; over 60% of patients with ST-elevation myocardial infarction (STEMI) present with multiple risk factors [30]. A systematic review further confirmed that multiple SMuRFs are especially common among young and middle-aged adults, significantly increasing their risk of both cardiovascular and cerebrovascular events [31].

Atherosclerotic cardiovascular disease poses a growing public health challenge in the Middle East, driven by increasing rates of obesity, diabetes, and hypertension. However, limited region-specific data exist on the distribution and impact of Standard Modifiable Risk Factors (SMuRFs) among affected individuals. Most available evidence is derived from Western populations, which may not reflect the unique demographic, cultural, and epidemiological profiles of Middle Eastern communities. By stratifying patients according to SMuRF burden, the study offers a novel framework for understanding how modifiable risk factors are associated with patient characteristics and care patterns in this underrepresented population. Given the growing cardiovascular disease burden in the Middle East and the lack of context-specific data, this research addresses a critical gap and supports the need for tailored, risk-based prevention and management strategies that reflect the unique demographic and epidemiological profile of the region.

Therefore, this study aimed to compare the demographic characteristics, clinical presentations, comorbidities, and pharmacologic management of Middle Eastern patients with atherosclerotic cardiovascular disease (ASCVD), stratified by the burden of standard modifiable risk factors (SMuRFs) into three groups: SMuRF-less (no risk factors), 1–2 SMuRFs, and 3–4 SMuRFs. The objective was to identify key differences across these groups. Specifically, the study examined variations in demographics, clinical features, and comorbidities among the different SMuRF categories. We hypothesized that comorbidities and pharmacologic management among Middle Eastern country (Jordan) patients with ASCVD would be higher according to higher levels of SMuRF.

## Methods

### Study design

The data presented in this study was drawn from two sources. The first source was a cohort of consecutive adult patients (age ≥ 18 years) diagnosed with ASCVD who were enrolled prospectively in the Jordan SMuRF-less Study (CliniclTrials.gov, identifier number NCT06199869) from January 10, 2024 through August 20, 2024 in three community hospitals and six tertiary care centers that included 3 ministry of Health hospitals, two university hospitals, and one teaching private hospital) in Jordan. The second source of the data was a post hoc analysis of patients with ASCVD enrolled in six Middle Eastern registries (Table 1) [32–38]. These registries are the First Jordan Percutaneous Coronary Intervention Registry (CliniclTrials.gov identifier NCT01841346) [32], the Atherosclerotic Cardiovascular Disease Novel and Classical Risk Factors in Young Middle Eastern Women Study (NCT04975503) [33], Surviving a Decade or More after Coronary Revascularization in a Middle Eastern Population Study (NCT03491722) [34], the Jordan Atrial Fibrillation Study (NCT03917992,18 Statin Eligibility Among Middle Eastern Patients Presenting with Acute Myocardial Infarction (NCT03485742) [36] and the Jordan Covid-19 Pandemic Acute Cardiovascular events Study (NCT04368637).20 Data were collected by trained coordinators using standardized case report forms. Demographic and anthropometric features, medical history, standard modifiable and non-modifiable and novel, nontraditional risk factors, comorbidities, utilization of pharmacotherapy for secondary cardiovascular prevention and one year survival after the first cardiovascular event were documented.

**Table 1:** Summary of Cardiovascular Studies and Registries in Jordan.

#	Study (ClinicalTrials.gov ID)	Design & Setting	Target Population	Main Objective/ Outcomes	Timeframe/ Status	Participating Sites
1	Jordan SMuRF-less Study (NCT06199869)	Prospective cohort, multiple hospitals in Jordan	Adults >=18 with ASCVD Excluded: CHD, pregnancy, no consent	Identify non-traditional ASCVD risk factors	Jan-Aug 2024	3 community + 6 tertiary centers (MoH, universities, private)
2	First Jordan PCI Registry (NCT01841346)	National PCI registry	Adults >=18 undergoing PCI Excluded: contraindications, severe co-morbidity	Evaluate PCI safety, complications, effectiveness	Ongoing	PCI-performing hospitals across Jordan
3	ASCVD Risk in Young Women (NCT04975503)	Cohort study, regional urban centers	Women 18–45, at risk or diagnosed ASCVD Excluded: pregnancy, prior major CV events	Identify novel vs classical ASCVD risks in women	Multi-year, ongoing	Urban centers across Middle East
4	Surviving 10 + Years Post-Revasc (NCT03491722)	Cohort study of long-term survivors post PCI/CABG	Survivors >=10 years post-revascularization Excluded: < 10y, terminal illness	Long-term survival, QOL after revascularization	Retrospective-ongoing	Hospitals across Middle East incl. Jordan
5	Jordan AF Study (NCT03917992)	AF registry in cardiology centers	Adults >=18 with AF Excluded: non-cardiac arrhythmia, pregnancy, no anticoag.	Assess AF prevalence, treatment, outcomes	Ongoing (since 2019)	Jordanian hospitals with arrhythmia care
6	Statin Eligibility in AMI (NCT03485742)	Registry of AMI cases	Adults with AMI eligible for statins Excluded: statin intolerance	Evaluate guideline adherence for statin use	Ongoing	High-volume AMI centers in Middle East
7	COVID-19 & Acute CV Events (NCT04368637)	Pandemic registry of acute CV events	Adults with MI, stroke during COVID Excluded: non-pandemic events	Impact of pandemic on CV emergencies	2020-present	Jordanian hospitals managing COVID + CV

The data were accessed for research purposes on May 28, 2024. All data used in the study were fully anonymized before access by the researchers. Patients were enrolled under serial numbers with no identifiable information, and as such, the Institutional Review Board (IRB) waived the requirement for obtaining individual informed consent for the secondary analysis of data from these registries.

### Inclusion criteria and definition of exposures

Patients with ASCVD included those with coronary artery disease (CAD), stroke, carotid artery disease and peripheral arterial disease. CAD patient included those with acute coronary syndrome (ACS) (ST-segment elevation myocardial infarction [STEMI] and non-ST-segment-elevation ACS), chronic coronary angina (CSA) and CAD diagnosed by coronary computed coronary tomography angiography (CCTA). Three groups of patients were studied; patients who were SMuRF-less, those with 1–2 SMuRFs, and those with 3–4 SMuRFs.

**Definition and Classification of Standard Modifiable Risk Factors** The SMuRFs were all defined as binary variables. Criteria of the diagnosis of HTN, type 2 diabetes (T2D), elevated serum LDL-C levels, and cigarette smoking was similar to those adopted by published studies [30, 38–42]. HTN diagnosis was defined as having a previous diagnosis by a treating physician, use of antihypertensive medications, or a new diagnosis during hospitalization with repeated measurements of systolic blood pressure ≥140 mm Hg and/or diastolic blood pressure ≥90 mm Hg. Type 2 diabetes was defined as a previous diagnosis, use of glucose-lowering medications, or serum level of hemoglobin A1c ≥ 6.5%. Dyslipidemia was inferred by a prior diagnosis of a treating physician, use of lipid-lowering agents, or elevated serum levels of LDL-C above the recommended target levels. A study participant was considered a current cigarette smoker in the presence of regular smoking within the past one year before enrolment.

### Definitions of other traditional risk factors

Two traditional risk factors were included in the analysis. Obesity was defined based on a body mass index ≥30 kg/m2. Positive family history of premature CVD was defined as the presence of a cardiovascular event in a first degree relative aged ≤55 years (male) or ≤65 year (female).

This non-interventional study was performed in accordance with the Declaration of Helsinki. The study received proper ethical oversight and Institutional Review Board approval from the participating institutions (Institutional Review Board/Independent Ethics Committee Istishari Hospital, Amman, Jordan). Each patient signed a written informed consent. The study is registered with ClinicalTrials.gov (NCT06199869).

The data analyzed in this study were derived from a cohort of 5,540 patients who had been enrolled in previously published cardiovascular research studies, all of which were registered on ClinicalTrials.gov. Informed consent was obtained from all participants at the time of enrollment, as detailed in the original study reports. For the purposes of this retrospective analysis, which utilized aggregated and fully de-identified data, the Institutional Review Board (IRB) approved a waiver of informed consent. This exemption was granted due to the absence of personally identifiable information and the considerable time elapsed since the original data collection, which rendered re-contacting participants impracticable.

### Statistical analysis

Data were analyzed using IBM SPSS Statistics version 24. Descriptive statistics were applied to summarize the sociodemographic, clinical, and medication-related characteristics of the sample (N = 5,540). Continuous variables were expressed as mean and standard deviation (M ± SD), whereas categorical variables were presented as frequencies and percentages (%). The research hypothesis (Comorbidities and pharmacologic management among Middle Eastern patients with ASCVD would be higher according to higher levels of SMuRF) which is the differences between the three groups—SMuRFS-Less (G1), one to two SMuRFS (G2), and three to four SMuRFS (G3)—was assessed using statistical tests. For continuous variables, one-way analysis of variance (ANOVA) was conducted, with Bonferroni post hoc tests applied when significant differences were found. Categorical data were analyzed with the chi-square (χ²) test. A p-value of less than 0.05 was deemed statistically significant for all analyses.

## Results

### Socio-demographic and clinical characteristics

The study sample (N = 5,540) with ASCVD was stratified into three groups based on SMuRF count: SMuRF-less (G1, n = 214), one to two SMuRFs (G2, n = 3,014), and three to four SMuRFs (G3, n = 2,312). As shown in Table 2, the mean age was 57.5 ± 11.6 years, with G3 being the oldest group (58.7 ± 10.6 years; *p* < 0.001). Most participants were male (75.9%), with no significant gender differences across groups. A stepwise increase in cardiovascular risk factors was observed with higher SMuRF burden: hypertension (0% in G1, 37.9% in G2, 88.9% in G3), diabetes (0%, 15.6%, 35.4%), and smoking (0%, 36.5%, 54.0%) (all *p* < 0.001). Family history of premature CVD also rose with SMuRF count (22.4%, 30.0%, 39.3%; *p* < 0.001), as did the prevalence of CKD (7.3%) and heart failure (15.1%) in G3 (*p* < 0.001 and *p* < 0.005). ACS was the most common diagnosis overall (83.7%), with a slightly lower rate in G1 (80.4%) than in G2 (84.8%) and G3 (82.4%). BMI increased with SMuRFs, from 26.9 ± 3.9 (G1) to 29.0 ± 4.9 kg/m² (G3; *p* < 0.001). Lipid profiles also worsened with higher SMuRF count: G1 had the lowest LDL cholesterol (95.2 ± 31.9 mg/dL) versus G2 (114.1 ± 45.2) and G3 (109.2 ± 45.6; *p* < 0.001), while G3 showed the highest total cholesterol and triglycerides and the lowest HDL cholesterol (*p* < 0.001 and *p* < 0.01).

**Table 2:** Sociodemographic and clinical characteristics of the sample ASCVD (N = 5540).

Variable	Total sample (N = 5540) N (%)	(G1): SMuRFS-Less (n = 214) n (%)	(G2) one to two SMuRFS (n = 3014) n (%)	(G3) three to four SMuRFS (n = 2312) n (%)	p value
Age, mean (SD)	57.5 ± 11.6	55.3 ± 12.8	56.7 ± 12.1	58.7 ± 10.6	<0.001
**Gender**					
Male	4207 (75.9)	165 (77.1)	2324 (77.1)	1718 (74.3)	NS
Female	1333 (24.1)	49(22.9)	690 (22.9)	594 (25.7)	
History of hypertension	3197 (57.7)	0 (0)	1141 (37.9)	2056 (88.9)	<0.001
History of diabetes Mellitus	2824 (51.0)	0 (0)	862 (15.6)	1962 (35.4)	<0.001
History of dyslipidemia	4053 (73.2)	0 (0)	1866 (61.9)	2187 (94.6)	<0.001
Smoking	2350 (42.4)	0(0)	1101(36.5)	1249 (54.0)	<0.001
Family history of premature CVD	1859 (33.6)	48 (22.4)	903 (30.0)	908 (39.3)	<0.001
History of CKD	316 (5.7)	6 (2.8)	141(4.7)	169 (7.3)	<0.001
History of Heart Failure	739 (13.3)	27 (12.6)	362 (12.0)	350 (15.1)	<0.005
**Diagnosis**					
ACS	4635 (83.7)	172 (80.4)	2557 (84.8)	1906 (82.4)	<0.001
CVA	254 (4.6)	13 (6.1)	128 (4.2)	113 (4.9)	
Chronic stable angina	651 (1.8)	29 913.6)	329 (10.9)	293 (5.3)	
BMI (kg/m^2^), mean (SD)	28.4 ± 4.8	26.9 ± 3.9	28.1 ± 4.8	29.0 ± 4.9	<0.001
LDL, mean (SD)	111.4 ± 45.2	95.2 ± 31.9	114.1 ± 45.2	109.2 ± 45.6	<0.001
Total cholesterol, mean (SD)	179.7 ± 52.1	162.4 ± 42.3	181.9 ± 50.6	178.2 ± 53.8	<0.001
Triglycerides, mean (SD)	184.8 ± 138.2	152.3 ± 110.3	169.8 ± 112.7	201.7 ± 159.7	0.001
HDL, mean (SD)	38.8 ± 14.6	41.7 ± 11.5	39.6 ± 11.9	37.9 ± 16.9	<0.01

ACS: Acute coronary syndrome, CKD: Chronic kidney disease, CVA: Cerebrovascular accident, CVD: Cardiovascular diseases.

Values are presented as M ± SD or number (%).

Table 3 presents medication use across the three ASCVD groups. Statins and aspirin were the most commonly prescribed drugs, with overall usage rates of 90.6% and 89.8%, respectively. Statin use showed a strong upward trend with increasing SMuRF burden: 67.8% in G1, 89.6% in G2, and 94.3% in G3 (*p* < 0.001). Aspirin followed a similar pattern—80.8% in G1, 89.4% in G2, and 92.3% in G3 (*p* < 0.001). Use of clopidogrel (Plavix) varied moderately among groups: 57.0% in G1, 53.8% in G2, and 58.1% in G3 (*p* < 0.01). Dual antiplatelet therapy (DAPT) was prescribed to 66.2% of the total cohort, increasing significantly from 61.7% in G1 to 69.4% in G3 (*p* < 0.001). Beta-blocker use also rose with SMuRF count—68.2% in G1, 71.7% in G2, and 76.6% in G3 (*p* < 0.001). P2Y12 inhibitor use was relatively low overall (14.3%) but significantly lower in G1 (8.4%) compared to G2 (14.2%) and G3 (15.0%) (*p* < 0.05). Use of oral hypoglycemic agents was strongly associated with SMuRF burden, ranging from 4.7% in G1 to 11.1% in G2 and 27.4% in G3 (*p* < 0.001), reflecting the increasing prevalence of diabetes in higher-risk groups.

**Table 3:** Comparisons of medication use among the three groups of ASCVD (N = 5540).

Medication used	Total sample (N = 5540) N (%)	(G1): SMuRFS-Less (n = 214) n (%)	(G2) one to two SMuRFS (n = 3014) n (%)	(G3) three to four SMuRFS (2312) n (%)	p value
Statins	5021 (90.6)	145 (67.8)	2696 (89.4)	2180 (94.3)	<0.001
Aspirin	5003 (90.3)	173 (80.8)	2695 (89.4)	2135 (92.3)	<0.001
Plavix	3088 (55.7)	122 (57.0)	1623 (53.8)	1343 (58.1)	<0.01
P2Y12 inhibitors	794 (14.3)	18 (8.4)	429 (14.2)	347 (15.0)	<0.05
Dual antiplatelet therapy	3665 (66.2)	132 (61.7)	1928 (64.0)	1605 (69.4)	<0.001
Beta blockers	4060 (73.3)	146 (68.2)	2142 (71.7)	1772 (76.6)	<0.001
Oral hypoglycemic agents	977 (17.6)	10 (4.7)	334 (11.1)	633 (27.4)	<0.001

## Discussion

This study examined 5,540 patients with atherosclerotic cardiovascular disease (ASCVD) from multiple registries across the Middle East. Participants were stratified into three groups based on the number of Standard Modifiable Risk Factors (SMuRFs): SMuRF-less (G1), one to two SMuRFs (G2), and three to four SMuRFs (G3). The analysis revealed significant and consistent patterns in demographics, comorbidities, clinical characteristics, and medication usage across these groups.

We found that patients with a higher number of SMuRFs were generally older. The mean age increased progressively across the three groups. This trend supports the well-established link between aging and cardiovascular disease and is consistent with previous global and regional studies identifying age as a major non-modifiable risk factor for ASCVD [16,43–45]. For instance, AlHabib, Sulaiman [46] reported a high prevalence of traditional cardiovascular risk factors especially hypertension and diabetes among individuals in the Gulf region, with these risks increasing further with age. In the Middle East, this relationship is especially significant, as rapid urbanization and lifestyle changes have led to rising rates of obesity, hypertension, and diabetes, particularly among older adults [44,47,48]. Similarly, this relationship is supported by international studies that have documented consistent patterns. For example, research from China shows that nearly 50% of older adults have some form of ASCVD, highlighting the substantial burden of cardiovascular disease in this population [49]. Additionally, Li, Gao (22) reported that older patients with STEMI tend to have a higher number of risk factors, which are associated with poorer outcomes.

Our study found that most participants were male, with no significant gender differences across groups. This finding is consistent with global trends indicating that men, on average, have a higher risk of cardiovascular disease compared to women. [50]. However, the lack of gender differences between groups suggests that while men are more commonly affected by ASCVD, modifiable risk factors impact both genders equally.

Our results showed that a stepwise increase in cardiovascular risk factors was observed with higher SMuRF burden: hypertension, diabetes, and smoking. This finding indicates a significant correlation between cardiovascular risk factors and the number of SMuRFs [38,51–54]. This pattern aligns with a systematic review showing a high prevalence of diabetes among patients with ACS in the Middle East, highlighting the need for targeted interventions [55,56]. Furthermore, dyslipidemia was highly prevalent in the total sample, with a notable increase in Group 3 compared to Group 1. This trend underscores the need for comprehensive lipid management strategies, particularly in patients with multiple risk factors. The high prevalence of dyslipidemia in the Middle Eastern population has been documented in various studies, indicating a pressing need for targeted interventions. Supporting this, a study by Alhyas, McKay (58) highlights that the prevalence of hypertension and dyslipidemia in the Gulf region is among the highest worldwide, driven by lifestyle changes and economic development. Specifically, 54% of patients in Group 3 were smokers, while none in Group 1 reported smoking. This aligns with global trends recognizing smoking as a key modifiable risk factor for cardiovascular morbidity and mortality [57].

The link between smoking and the accumulation of cardiovascular risk factors is well established, reinforcing the need for targeted smoking cessation interventions to reduce ASCVD risk, especially among the elderly. In the Middle East, smoking remains a major public health challenge [58]. A study by Rauf, Khan [59] found that smoking, along with other modifiable lifestyle risks like hypertension and diabetes, significantly contributes to the projected future burden of cardiovascular events in the region.

Additional clinical risks, such as chronic kidney disease (CKD) and heart failure, were also more common among patients with a higher SMuRF burden. In G3, CKD affected 7.3% of patients, and heart failure 15.1%. This indicates that accumulating SMuRFs not only elevates traditional cardiovascular risk but also increases the likelihood of developing serious end-organ complications [60–62]. A family history of premature cardiovascular disease was also more frequently reported in higher-risk groups, rising from 22.4% in G1 to 39.3% in G3 (p < 0.001), underscoring the role of genetic predisposition in ASCVD development [63].

In terms of clinical presentation, acute coronary syndrome (ACS) was the most common diagnosis across all groups. However, it was slightly less frequent in the SMuRF-less group compared to G2 and G3. This suggests that although SMuRF-less individuals can still develop ASCVD, their clinical profiles may differ. Body mass index (BMI) also increased with SMuRF burden, rising from 26.9 kg/m² in G1 to 29.0 kg/m² in G3. Similarly, adverse lipid profiles including elevated total cholesterol and triglycerides, and reduced HDL cholesterol were most pronounced in G3. These trends highlight the metabolic complexity of high-risk individuals and the need for earlier preventive interventions [64].

Medication use varied significantly across the groups. Statin and aspirin use increased progressively from the lowest to the highest SMuRF burden group, suggesting that patients with a greater cardiovascular risk are more likely to receive guideline-directed medical therapies [38,65–67]. Similarly, the use of beta-blockers and dual antiplatelet therapy (DAPT) was more frequent among those with higher SMuRF levels. Although the overall use of P2Y12 inhibitors was relatively limited, their prescription was more common in patients with greater SMuRF burden (p < 0.05). These trends highlight a pattern of more intensive pharmacologic management in individuals with elevated cardiovascular risk. [38,67,68]. Interestingly, the use of clopidogrel (Plavix) remained relatively consistent across all SMuRF groups, suggesting that its prescription may be less influenced by individual risk burden compared to other cardiovascular therapies. This observation aligns with findings from the CHARISMA trial and other studies, which demonstrated no significant benefit of adding clopidogrel to aspirin for primary prevention in patients with multiple risk factors.

[41,69–71]. In contrast, the use of oral hypoglycemic agents increased substantially across the SMuRF burden groups, reflecting a greater emphasis on glycemic control in patients with higher cardiovascular risk. This pattern underscores the role of diabetes management as an integral component of comprehensive cardiovascular risk reduction, particularly in high-risk populations. [72,73].

In summary, patients with a higher SMuRF burden were older, had more comorbid conditions, demonstrated worse metabolic profiles, and were more likely to receive intensive pharmacologic treatment. These consistent trends across SMuRF groups underscore the need for comprehensive and individualized risk assessment and management strategies in Middle Eastern populations. Tailored interventions for patients with multiple risk factors are essential to reduce the long-term burden of ASCVD and improve clinical outcomes.

Clinically, this study emphasizes the urgent need for early identification and aggressive risk management, particularly in individuals with multiple SMuRFs. As ASCVD rates continue to rise in the Middle East, strategies must extend beyond treating existing disease to include robust public health efforts focused on smoking cessation, nutrition, diabetes management, and physical activity. From a research perspective, larger and more diverse cohorts are needed to enhance generalizability, along with long-term studies to evaluate outcomes and treatment effectiveness. Future work should also explore the integration of digital health tools, emerging pharmacologic therapies, and policies that improve healthcare access. Together, these approaches can help reduce the escalating burden of ASCVD in the region.

In summary, patients with a higher SMuRF burden were older, had more comorbid conditions, demonstrated worse metabolic profiles, and were more likely to receive intensive pharmacologic treatment. These consistent trends across SMuRF groups underscore the need for comprehensive and individualized risk assessment and management strategies in Middle Eastern populations. Tailored interventions for patients with multiple risk factors are essential to reduce the long-term burden of ASCVD and improve clinical outcomes.

Clinically, this study emphasizes the urgent need for early identification and aggressive risk management, particularly in individuals with multiple SMuRFs. As ASCVD rates continue to rise in the Middle East, strategies must extend beyond treating existing disease to include robust public health efforts focused on smoking cessation, nutrition, diabetes management, and physical activity. From a research perspective, larger and more diverse cohorts are needed to enhance generalizability, along with long-term studies to evaluate outcomes and treatment effectiveness. Future work should also explore the integration of digital health tools, emerging pharmacologic therapies, and policies that improve healthcare access. Together, these approaches can help reduce the escalating burden of ASCVD in the region.

## Strengths and limitations of the study

A key strength of this study is the rigorous approach taken to harmonize data from multiple registries across the Middle East, despite their varying designs and data collection methods. Standardized case report forms were used across all registries to ensure consistent collection of demographic, clinical, and pharmacologic information. Uniform definitions for standard modifiable risk factors (SMuRFs) and clinical outcomes were applied to enhance comparability. Comprehensive data cleaning and a post hoc analysis assessing variability across registries further minimized potential bias and heterogeneity. These measures increase the reliability and robustness of our findings, providing a solid foundation for understanding ASCVD risk factors in this diverse population.

This study has several limitations. Although the sample size was relatively large, comprising over 5,500 patients, all data were derived from a single Middle Eastern country. As such, the findings may not be fully generalizable to the broader Middle East region, which is diverse in terms of population demographics, healthcare systems, and disease patterns. Future studies involving multicenter and multinational cohorts are warranted to confirm and extend these findings across a more representative Middle Eastern population. Another limitation of this study is the use of relatively simple statistical analyses. While these methods were appropriate for addressing the primary objective and the secondary hypothesis, they may not fully capture the complexity of relationships among variables. More advanced statistical modeling could provide additional insights and should be considered in future research to strengthen the robustness and depth of analysis.

## Conclusion

This study reveals notable variations in socio-demographic characteristics, clinical presentations, comorbidities, and medication use among Middle Eastern patients with atherosclerotic cardiovascular disease (ASCVD), grouped by the number of modifiable risk factors (SMuRFS). As the number of SMuRFS increases, patients tend to be older and show higher incidences of hypertension, diabetes, dyslipidemia, and require more intensive medication regimens, including statins, aspirin, and beta-blockers. In the group with the highest number of SMuRFS (G3), patients exhibited the most severe clinical conditions, including chronic kidney disease, heart failure, and poor lipid profiles. Diabetes management was particularly important, with a significant increase in the use of oral hypoglycemic agents in this group. These findings highlight the need for tailored treatment strategies that address each patient’s unique risk factors, especially in regions experiencing rising cardiovascular disease rates due to urbanization and lifestyle changes. The study highlights the necessity of focused interventions and aggressive pharmacological treatment to improve patient outcomes and prevent further progression of ASCVD.

## Supporting information

S1 Data Dataset. (XLSX)
